# ‘I don’t know what normal has been’: a grounded theory exploration of the journey to endometriosis diagnosis

**DOI:** 10.1186/s12905-025-03869-y

**Published:** 2025-07-04

**Authors:** Babu Karavadra, Joanna Semlyen, Edward Morris, Gabrielle Thorpe

**Affiliations:** 1 University of East Anglia, Norwich Medical School Norwich Research Park, Norwich, Norwich, NR4 7TJ UK; 2 Obstetrics & Gynaecology, Norfolk & Norwich University Hospital, Colney Lane, Norwich, NR4 7UY UK; 3 University of East Anglia, School of Health Sciences, Norwich Research Park, Norwich, NR4 7TJ UK

**Keywords:** Endometriosis, Delay, Diagnosis, Grounded theory, Qualitative

## Abstract

**Background:**

Diagnosis of endometriosis in the United Kingdom takes on average eight years, with delay to diagnosis contributing to physical, psychological and social burden for women experiencing endometriosis. This study aimed to explore experiences of diagnosis in women with confirmed endometriosis.

**Methods:**

The study was informed by Constructivist Grounded Theory. Purposeful sampling was used to recruit fifteen women with confirmed endometriosis to participate in semi-structured interviews. Delay to diagnosis was identified as a key finding after analysis of four interviews and was therefore a focus for subsequent theoretical sampling. Constant comparative analysis generated codes and categories and ultimately a draft theory.

**Results:**

A novel theoretical framework was developed, illustrating how participants fluctuated through four contexts of refusal, strong disbelief, weak disbelief and belief in their diagnosis journey, underpinned by a core category of ‘making sense of a fluctuating life’. Within each context, the framework explicates how relational power and self-perception engenders a strong psychosocial influence on recognition of risk of harm from symptoms of endometriosis and consequent investigating behaviour.

**Conclusions:**

The journey to diagnosis of endometriosis involves a complex interplay of psychological, social and relational factors in driving or inhibiting help-seeking behaviour, requiring sensitivity, understanding and a commitment to listen and value women’s experiences within the clinical consultation to ensure timely and appropriate investigation and management.

**Supplementary Information:**

The online version contains supplementary material available at 10.1186/s12905-025-03869-y.

## Background

Endometriosis is a common condition in women wherein endometrial stromal cells are found outside the endometrium (Giudice & Kao, 2004). The most common locations for endometriosis to develop include the ovaries, fallopian tubes and pelvis (Nisolle & Donnez, 2019). Endometriosis can also develop within the gastrointestinal tract and the urological system, resulting in dyschezia (difficulty in passing stool), blood in the stool and/or urine, and difficulties with urinating (Nisolle & Donnez, 2019). Although traditionally viewed as a pelvic disease, endometriosis is now increasingly recognised as a chronic systemic disease that extends beyond the pelvis, including the alteration of gene expression and provoking systemic inflammation (Taylor et al., 2021). As endometriosis can occur in young women aged 17 years and younger (National Institute for Health & Care Excellence (NICE), 2024), the terms ‘women’ and ‘woman’ used throughout this paper are inclusive of adolescent girls as well as women of 18 years and older when applied in general terms to all women who menstruate.

The ways in which endometriosis impacts on quality of life is well described in the literature (Lightbourne, 2024; [25]). In a qualitative interview-based study of 16 women with endometriosis, Roomaney and Kagee (2016) found that symptoms associated with endometriosis had a negative impact on work productivity, with consequent financial implications, as well as on physical, psychological and sexual function,many women felt impaired in performing daily activities that were taken for granted, described feelings of isolation and hopelessness, and experienced painful sexual intercourse, resulting in relationship tensions (Roomaney & Kagee, 2016). Relationship tensions can be compounded by subfertility associated with endometriosis (Moss et al., 2021). These findings resonate with a longitudinal case-controlled study of 567 women under 25 years of age, in which Gallagher et al. (2018) found that young women with endometriosis had worse physical and mental component scores when compared with controls, clearly highlighting the negative impact of the condition on quality of life. In addition, the longer the time to diagnosis, the more significant the impact of endometriosis was found to be on quality of life (Gallagher et al., 2018).

Delay to diagnosis of endometriosis is common. In the UK, it can take up to ten years to diagnose the condition (NICE, 2024; [7],Agarwal et al., 2019 and All Party Parliamentary Group (APPG) on Endometriosis, 2020). Indeed, endometriosis has been termed ‘the missed disease’ and has an influence on the women’s health gap (The Lancet, 2024). In a systematic review of 13 qualitative studies, Davenport et al. [12] identified individual factors, interpersonal influences, health system factors and factors specific to endometriosis as key barriers to the diagnosis of endometriosis. In 2017 (updated in 2024), NICE published guidelines on the diagnosis and management of endometriosis, making reference to the timeframe involved in diagnosis as a research priority. The guideline highlighted a need to improve the diagnosis of endometriosis and to explore the most effective ways to educate healthcare professionals throughout the healthcare system to facilitate a reduction in time to diagnosis (NICE, 2017,updated 2024).

A timely diagnosis for any medical condition is important; in endometriosis, a diagnosis facilitates consideration of appropriate medical and surgical treatment options to improve health-related quality of life. A diagnosis helps women to understand and legitimise their symptoms, supporting informed decision-making (Gaedtke, 2023). For those with subfertility, a diagnosis prior to commencing artificial reproductive treatment enhances the likelihood of conception (Moss et al., 2021).

The aim of this study was to explore the experiences of women with diagnosed endometriosis to understand factors influencing diagnosis in women with endometriosis and how these impact on their journey to diagnosis.

## Methods

### Study design

This study was informed by constructivist grounded theory (CGT) [9] to identify an underlying theoretical framework to elucidate the factors that influence the experience and process of diagnosis of endometriosis and how they interact. CGT assumes that knowledge is constructed, rather than simply discovered [9] and is underpinned by symbolic interactionism. Symbolic interactionism enables insights to be gained about how people make sense of the world by identifying, exploring and interpreting the meaning of the actions and interactions (constructs) that individuals have with society [8]. Consistent with CGT methodology, this study began with an exploration of experiences of diagnosis of endometriosis and allowed a theory of delay to diagnosis to develop from the inductive analysis of the data [9].

The research site for this study was a large NHS teaching hospital trust the UK. Four women with endometriosis who were not participants in the study, two doctors (a consultant gynaecologist and a registrar in obstetrics and gynaecology who was training to become a consultant) and a sociologist comprised the study team and were involved in the design of the study. The study was approved by the London-Surrey Borders Research Ethics Committee via the Integrated Research Application System (IRAS) (approval no. 223380) and conducted in accordance with the ethical principles outlined in the Declaration of Helsinki. All participants were fully informed about the study through a participant information sheet and an opportunity to discuss any questions before providing written consent prior to enrolment in the study.

### Sampling and Recruitment

Purposeful sampling was used to identify people meeting the eligibility criteria. Those eligible for inclusion in the study were women aged 18 years or over with a confirmed histological diagnosis of endometriosis, able to converse in English and with mental capacity to provide informed consent. There were no exclusion criteria. The study was advertised in the gynaecology outpatient department of a tertiary referral centre as well as via the local radio station, clearly defining the purpose of the research and the eligibility criteria. Snowball sampling, through which participants informed other women with endometriosis about the study, also facilitated identification of ‘hidden populations’ of women, who may not have had direct access to the gynaecology clinic (Dragan & Isaic-Maniu, 2013).

### Data collection

Fifteen women with confirmed endometriosis participated in the study (Table 1) between March 2018 to September 2018. Semi-structured interviews were conducted by BK using an interview schedule (Supplementary File 1) co-constructed with the study team. Interviews were all conducted in person, lasted between 30 and 120 min and took place in a private room in the Gynaecology department at the research site. Each interview was audio recorded and transcribed verbatim, with participants assigned a pseudonym at transcription.

**Table 1:** Participant demographics

Participant pseudonym	Age	Race	Time to diagnosis from onset of symptoms (years)
Janet	22	White British	2
Diana	23	White British	5
Gemma	45	White British	23
Anna	22	White British	4
Harissa	34	Asian Indian	1.5
Umbola	43	Black African	17
Barbara	28	White British	1
Faye	27	White British	5
Neha	23	Asian British	4
Millie	40	White British	5
Alma	22	White British	5
Bonnie	42	White British	4
Elisha	28	Asian Mixed	3
Erika	32	White British	3
Vivienne	33	White British	4

As a male registrar in obstetrics and gynaecology at the time, BK was interviewed by a qualitative researcher to draw out any unconscious pre-conceptions that might influence his stance during data collection. During interviews, active listening and open posture were used to establish rapport. The initial open question ‘please can you take me through your journey of being diagnosed with endometriosis?’ allowed participants, rather than BK, to guide the conversation, with further open questions and prompts used to explore meaning and to deepen understanding.

Following analysis of the first four interviews, it became clear that delay to diagnosis was a consistent focus in participants’ experiences. Women who specifically experienced a delay to diagnosis of endometriosis were therefore theoretically sampled to explore this aspect of the experience of diagnosis in greater depth [11]. Concurrent data collection and constant comparative data analysis [9, 24] were informed by memo writing to capture emerging thoughts and ideas which influenced theoretical sampling and interpretations within the coding process. Theoretical saturation refers to the point at which no new properties are generated for the chosen core categories [9] and was achieved after 15 interviews.

### Data analysis

**Fig. 1:**
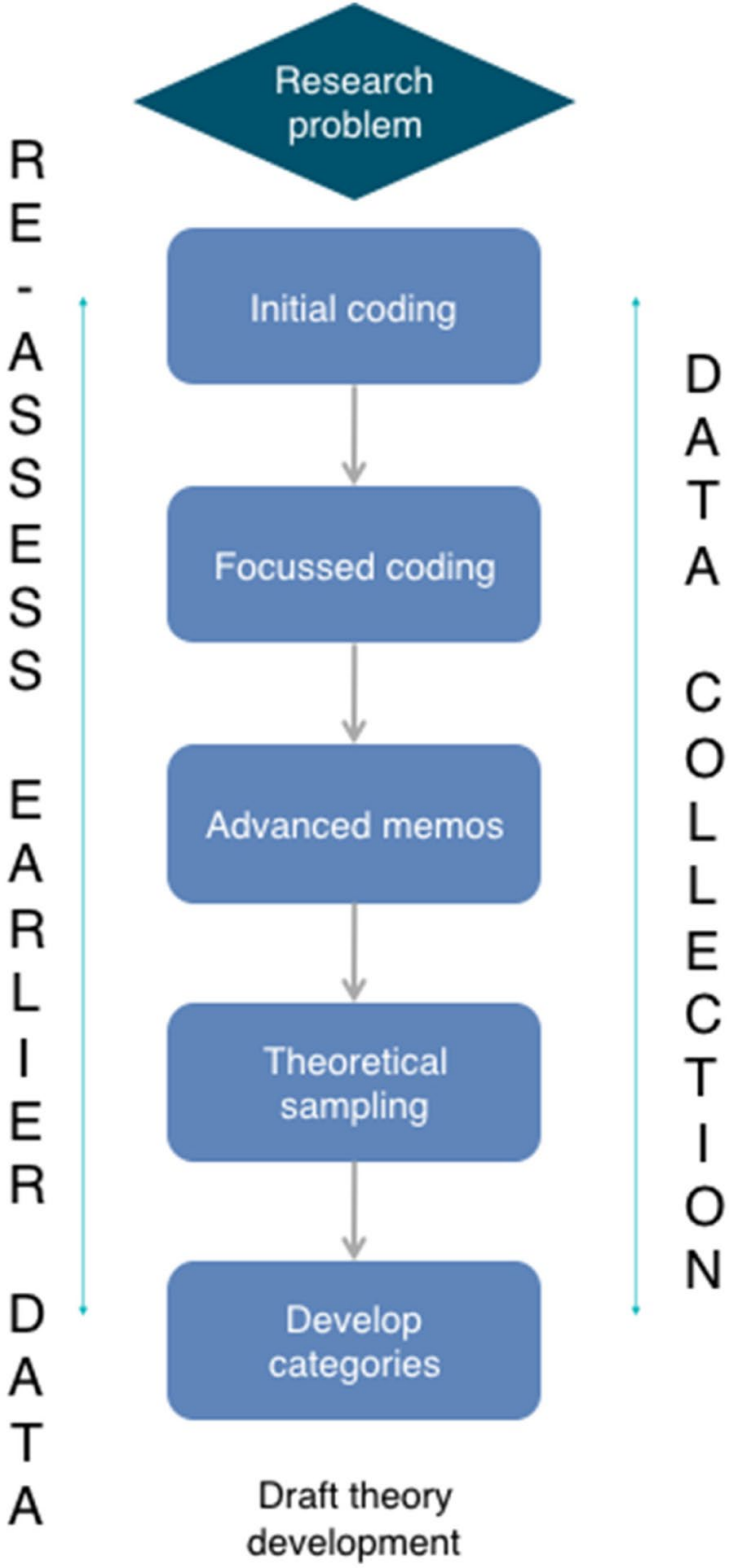
adapted (re-created) overview of the process involved in developing a theory, as envisaged by Charmaz (2006, p.11)

Constant comparative analysis was applied in coding and category development, involving iteration back to raw data and across data sets to test impressions and interpretations and then forwards to generating codes and categories and ultimately identifying a draft theory [9]. Figure 1 shows a schematic overview of the overall process involved in generating a CGT, as depicted by Charmaz (2006, p.11).

Initial coding is used in CGT to identify, compare, label and connect ‘incidents’ (Tie et al., 2019) – words or phrases that appear meaningful within the data. In keeping with CGT methodology, code labels were grounded in the participants’ words [9]. Codes and emerging categories highlighted ‘clues’ that could be further explored in concurrent data collection [5] and were discussed within the research team to ensure that interpretations made were grounded in the data rather than influenced by preconceived notions. Focused coding was used to inform a process of abstraction, through which categories were grouped around a potential core category, with theoretical coding then used to synthesise categories into a conceptually coherent and organised theory (Tie et al., 2019). A ‘storyline’ approach was used to present and explain the final Grounded Theory, creating a narrative of diagnosis of endometriosis that connected categories and elucidated the complexity underpinning delay to diagnosis (Birk & Mills, 2011). Summarising concepts used to frame the final Grounded Theory were generated through reflective interpretation with key quotes used to ‘ground’ theoretical coding.

## Results

The underlying issue for women with endometriosis identified in this CGT study involves ‘making sense of a fluctuating life’ (the core category). The study highlighted physical and psychological symptoms experienced by women with endometriosis impact on every aspect of their lives, influencing their experiences of work and education and particularly impacting on their close relationships with family and friends. Critically, women’s experiences of the illness itself, and of their attempts to make sense of their symptoms, have a profound and negative psychological impact, especially with regards to their own identity. As their lives fluctuate externally and internally, women seek to make sense of their experiences by exploring and investigating their symptoms, which leads them, sooner or later, to seek a diagnosis.

### Contexts

**Fig. 2:**
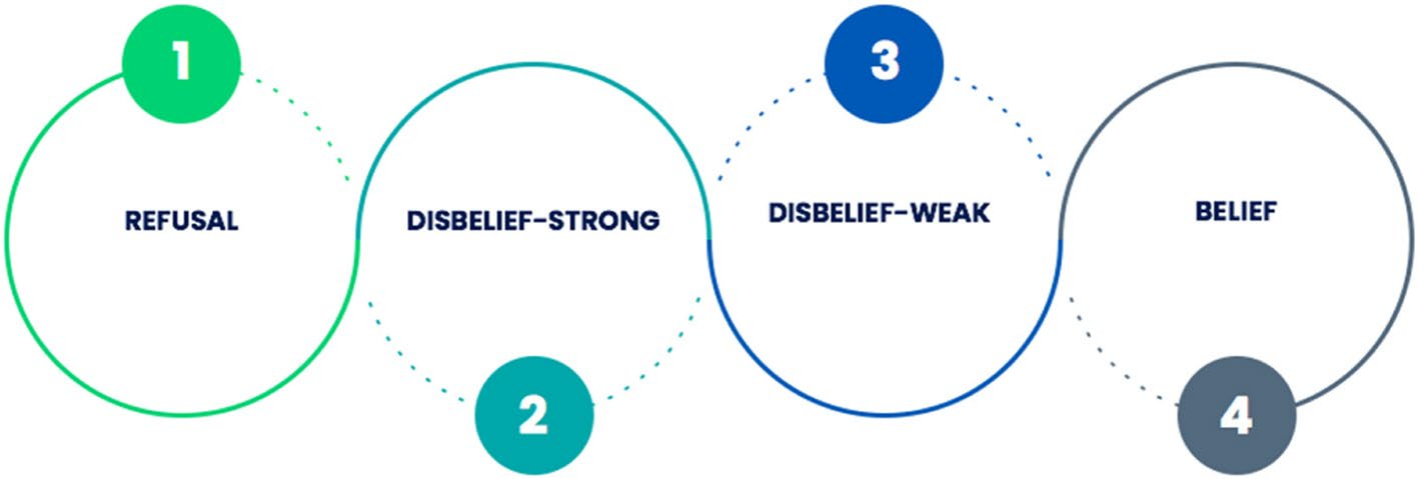
Diagrammatic representation of the women’s contexts in their experience of diagnosis of endometriosis

Women’s pathways to diagnosis of endometriosis are likely to involve four ‘contexts’: refusal, disbelief (stronger and weaker) and belief (Fig. 2). The word context is used in this study to mean a psychological perspective which influences a woman’s beliefs about their symptoms and themselves and their help-seeking behaviour. The contexts through which each woman experiences her diagnosis are relational. This means that each woman cycles between different contexts, sometimes fluctuating back and forth over time.

In the refusal context, the woman does not display overt help seeking behaviour and therefore diagnosis is highly unlikely. In the strong disbelief context, the woman will begin to recognise her symptoms as abnormal or problematic, but often indirectly through the disruptive impact they have on her life in general; the woman may therefore lack the insight or confidence to challenge cultural norms and is likely to test her perception of abnormality with trusted others rather than through seeking professional help. In the weak disbelief context, the woman will feel more secure in her belief of the abnormality of her symptoms and is likely to seek professional help, although formal diagnosis within this context remains unlikely due to her lack of self-confidence and the perceived low credibility of her evidence by healthcare professionals. In the belief context, the woman's investigating behaviours and the clinician’s response to them is most likely to lead to a diagnosis.

**Fig. 3:**
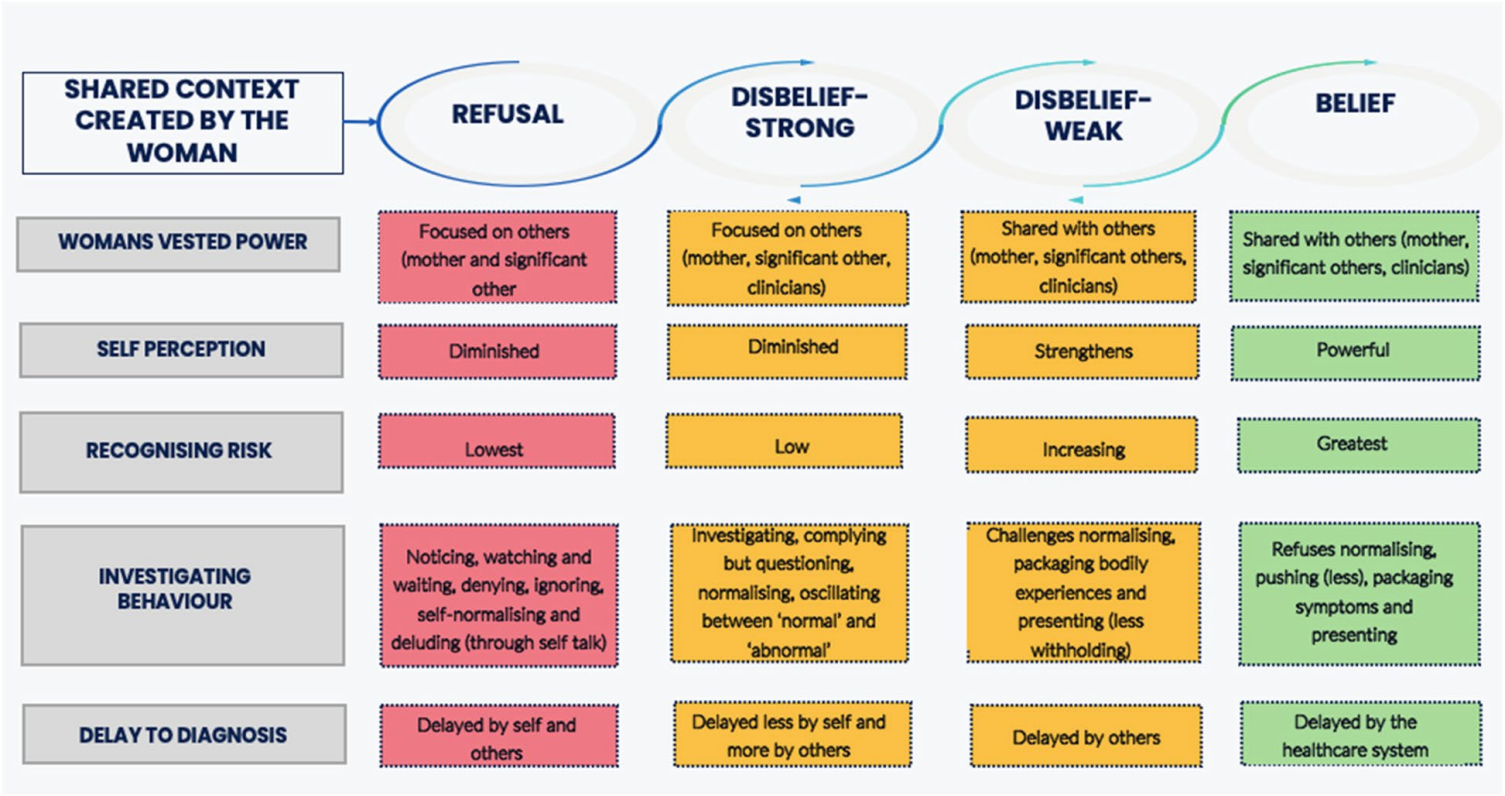
The grounded theory: Different contexts and their influencing factors

Grounded in participants’ experiences, our findings highlight how a woman’s context is influenced by (i) the way in which other people respond to her and the balance of power within these relationships (ii) how each woman perceives herself (iii) the extent of risk that the woman recognises in relation to her symptoms and the impact these have on her life, and (iv) how the previous three factors influence her investigating behaviour. These factors work together to influence the way a woman interprets the meaning of her symptoms of endometriosis and therefore the way in which she accesses information and support to address them through help-seeking behaviour, ultimately impacting on the time taken to diagnose endometriosis (Fig. 3).

The four contexts form a framework through which the findings of this study are represented, with participants’ experiences of relational power, self-perception, recognition of symptom risk and investigating behaviour explored within each context and together used to explain how these can influence a woman’s experiences and the duration of the process of diagnosis of endometriosis.

### Context of refusal: “It’s normal”

The context of refusal is characterised by a woman not recognising that she has a problem for which she needs to seek help. She is likely to be heavily influenced by those in positions of power in her life, who may not recognise that she has a problem and tend to reassure her that her experiences are merely normal. This can diminish a woman’s autonomy as those around her may portray help seeking behaviour as unnecessary, embarrassing or even attention seeking and reinforce feelings of shame and a perception of stigma regarding her symptoms, which they believe should remain hidden. This ‘normalisation’ or dismissal of symptoms means that a woman does not recognise that she has a problem for which she might need to seek support and help from others, particularly healthcare professionals, meaning that diagnosis of endometriosis is almost impossible, unless identified incidentally.

### Power: “I was told it’s women’s problems and it’s normal”

When describing their experiences within the refusal context, participants revealed that power within relationships tended to be focussed on others, most commonly the woman’s mother, partner or spouse. When participants sought to ‘check out’ their symptoms with people they trusted, the symptoms tended to be normalised or dismissed, sometimes making the woman feel that she was exaggerating or making a fuss. As many participants first experienced their symptoms when they were an adolescent and had no prior normal menstrual experiences to compare their symptoms to, they placed their trust in the people who they thought would know better and therefore did not question the responses they received.

‘My parents knew how much pain I was in, but they said it will become better in time and I just went with that’ (Elisha).

When symptoms related to participants’ menstrual cycle, their mothers often normalised her experiences, citing a family history of heavy periods or gynaecological issues.

‘Mum always said my nan’s periods were bad. It gives the impression that maybe this is just how women in my family are built’ (Faye)

‘I was told it’s women’s problems and it’s normal. Gynae problems run in my family and so I always knew this was normal for me. My mum even told me they were normal for us all’ (Millie).

When Alma sought advice about her heavy and painful periods from her school nurse, she was reassured that they were normal and that she need not worry.

‘Even at school, my periods were so heavy and so painful. But the school nurse said they were fine. So then, I thought it was normal too.’ (Alma).

### Self-perception: “I am weak and pathetic because I can’t deal with periods”

In the context of disbelief, some participants described a need or a perceived or actual expectation from others to hide or ignore their endometriosis symptoms, by their nature intrinsically bound to their identity as a woman, which therefore created a tension in their self-perception. Bonnie shared these feelings in relation to her relationship with her boyfriend, while Alma referred to her encounter with the school nurse, both demonstrating that others’ dismissal of their symptoms led to feelings of stigma and shame and on their recognition of the severity and meaning of their symptoms:

‘I just couldn’t believe it when my boyfriend asked me to stop talking about my period and said it was weird to talk about it. It made me feel awkward and that I shouldn’t talk about it’ (Bonnie).

‘I learnt very quickly at age 11 that you can’t talk about periods. The school nurse tells you not to talk about them or doesn’t want to hear about it... everyone has periods and I felt that I was weak and pathetic because I can’t deal with them’ (Alma).

### Recognising risk: “periods are supposed to be painful and heavy”

All participants discussed how in retrospect they could recognise when they started experiencing abnormal bodily experiences, but these were not recognised as problems or ‘symptoms’ at the time:

‘I don’t know what normal has been because from the very first period I ever had was heavy and its always been really heavy, it’s always been really, really painful, but I suppose in my teens I just put that down to what I expected of a period, it was supposed to be painful, it was supposed to be heavy, you know, that’s what I thought was normal’ (Vivienne).

### Investigating behaviour: “it didn’t even cross my mind to see a doctor”

In the context of refusal, participants did not recognise risk and so any investigating behaviour was limited to seeking reassurance from trusted others.

‘I never thought I would have a gynaecology problem like this. When I first felt unwell, I kept talking to my mum and got through it. It didn’t even cross my mind to see a doctor’ (Erika)

Normalisation of their symptoms by participants themselves also meant that they were not inclined to seek medical help in this context.

‘I genuinely thought heavy periods were normal because everyone else around you makes you feel that. You don’t even think to see a doctor when you are told your symptoms are just normal’ (Faye)

#### Context of strong disbelief: “I knew there was a problem … I just blanked it out of my mind”

A woman leaves the refusal context and moves to the context of strong disbelief when she recognises that there is a problem. In a context of strong disbelief, a woman consciously notices her bodily experiences as abnormal and starts to explore their meaning. This context is characterised by narratives such as ‘watching and waiting’ ‘sense-making’ and ‘testing ideas and perceptions of the problem with others.’ However, in this context, a woman remains strongly influenced by others’ opinions and will therefore be persuaded to doubt her own perception of risk as her symptoms continue to be normalised. As a result, she may internalise feelings of shame in relation to her abnormal symptoms and perceive herself as abnormal rather than recognising her symptoms as worthy of investigation, affecting her self-perception. Women still do not seek healthcare professional support in this context as they are unlikely to perceive their symptoms as a medical problem. For these reasons, a woman is very unlikely to be diagnosed with endometriosis in a context of strong disbelief.

### Power: “I trusted him more than I trusted myself”

In the context of strong disbelief, power can be focussed on a significant other, such as a parent, partner or spouse. Erika illustrates here how her partner’s response strongly influenced how she made sense of her symptoms:

‘I wanted it all to go away. I knew there was a problem. My partner told me that the pain will get better and just to wait. I just blanked it out in my mind for a bit despite feeling so awful and in pain all the time. I guess I just trusted him more than I trusted myself’ (Erika).

Umbola describes a sense of resignation that even though she recognised she had a problem, the responses of others made her feel uncomfortable talking about it and therefore disempowered her sense of agency to seek the help she needed.

‘What’s the point in even talking about how painful my periods were when everyone makes you feel awkward about and tells you it is part of being a woman. In hindsight, I wish I had the confidence to speak up’ (Umbola)

### Self-perception: “I went through a phase of believing it was normal and then abnormal”

When participants in this study recognised that they had a problem, many described ‘watching and waiting’ or trying to ignore their symptoms for fear that recognising their abnormality would mean changes in their life for which they were not ready. As illustrated above and in this quotation from Alma, the power of the partner can impact strongly on the woman's self-perception, making her doubt herself and the significance of her symptoms.

‘I was like, well why are all these test results coming back as normal. Before I went to the doctor, I went through a phase of believing it was normal and then abnormal. Mum made me think it was abnormal. After the scan came back normal, I thought my symptoms probably are just normal and there is nothing wrong. I then didn’t see the doctor for a while’ (Erika).

### Recognising risk: “everything I was going through was not normal”

In this context, a woman is likely to be in the early stages of her illness and seeks to make sense of what is happening. As part of their sense-making, some participants described trying to compare their bodily experiences to their first experiences of menarche and because the bleeding and pain were intense then, they tended to normalise their subsequent symptoms.

‘As I got older, I remember comparing how bad the pain was to my first period- it was so painful from the start and so this was just normal for me didn’t feel the need to see the GP’ (Umbola)

Some did not recognise risk in terms of symptoms but described other aspects of their lives that were impacted negatively (‘fluctuating’) by their symptoms, which highlighted that there was a problem. One participant recognised her bodily experiences were abnormal when she overheard her parents argue about the amount of time she was taking off school as a result of her period pains. Their apparent acknowledgement of her symptoms enabled her to recognise herself that she had a problem.

‘I remember one day my parents arguing about how much time I had off school. It was at this point that I started to think that everything I was going through was not normal- not that this made me go to the doctor straight away!’ (Elisha)

As well as the impact of their symptoms on their relationships, indirect signs of a fluctuating life triggered by their symptoms of endometriosis were reflected in participants’ accounts of how they curtailed their social activities, being unable to continue swimming (Janet), running (Neha), playing hockey (Diana) or participating in physical education classes (Faye). This had an emotional and psychological impact on some women.

‘I remember telling my partner that my periods were getting so bad and heavy and it was making me depressed’ (Elisha)

### Investigating behaviour: “I waited for so many months”

In a context of strong disbelief, investigating behaviour appeared to continue to be influenced by participants adopting a ‘watch and wait’ approach and a general sense that the credibility of their evidence is too weak to present it to a healthcare professional.

‘Before I even went to see them [GP], I waited for so many months as I thought things would get better’ (Alma).

I eventually went and saw the GP, but this took months on end before I did’ (Neha).

Janet demonstrates how interactions with powerful others can have a profound effect on investigating behaviour in the context of strong disbelief. In the face of the normalising behaviour of her mother, she oscillated between telling herself that her experiences were normal, despite recognising the severity of her pain. This acceptance of her mother’s normalisation led Janet to suspend her investigating behaviour.

‘When I look back at my younger self, why did I think my symptoms were normal for so long? I know for sure that my mum was a big part in this as she tried to reassure me that the pain will go away. I just accepted it, for years literally’ (Janet)

#### Context of weak disbelief: “I knew my symptoms were not normal … I challenged her [GP]”

In the context of weak disbelief, the woman is more confident in her recognition of risk of harm from her symptoms and therefore more empowered to argue against symptom normalisation or dismissal from others. The woman is persistent and tenacious in pursuing health care professional help through her investigating behaviour. How healthcare professionals respond to a woman’s investigating behaviour largely determines whether the woman will oscillate back to a context of strong disbelief (if they dismiss symptoms as normal or not worthy of investigation) or move forward to belief (by recognising that there is a problem and as such, start to undertake a range of investigations). A diagnosis is more likely in this context.

### Power: “I changed to a different GP who didn’t normalise my symptoms”

In this context, participants were sufficiently empowered to seek help for their symptoms, but some found that clinicians did not recognise the symptoms and experiences that participants described as important or credible. Participants responded in one of two ways when this happened, either accepting this assessment and moving back to a context of strong disbelief or challenging this response. Alma and Umbola demonstrated in their narratives how the balance of power had changed at this stage from earlier in their journey to diagnosis – they were confident in the abnormality of their symptoms, which empowered them to challenge their GP’s decision-making:

‘When I saw the GP and explained my symptoms, he said this was normal and there was no need for a gynaecology referral. I got angry and asked why he refused to do so’ (Alma).

‘I knew my symptoms were not normal and when the GP said there wasn’t anything wrong with me, I challenged her. It was a weird feeling at first, but I did it. I eventually changed to a different GP who didn’t normalise my symptoms’ (Umbola)

In this context, the healthcare professional may recognise that there are symptoms to be treated but may not recognise the risk or significance of the problem, so frustrating the woman’s attempts to have their symptoms investigated further and gain a diagnosis. Gemma expressed her frustration at her GP’s response to her help-seeking behaviour:

‘A lot of GPs treat the symptoms rather than the actual cause. I know it’s hard, but surely they can treat the symptoms and investigate you for why it’s actually happening. Just starting the pill is not enough!’ (Gemma).

For Alma, although her recognition of her abnormal bodily experiences drove her to seek medical help, the dismissal of her symptoms as normal by two professionals in positions of authority made her question the credibility of her own evidence and instigated a move backwards to a context of strong disbelief.

‘I saw a gynaecologist there and my family doctor - they both reassured me it’s normal. When you have a professional like a doctor and someone close to you like your family telling you its normal, then what am I supposed to believe? Of course, I accepted this!’ (Alma).

### Self-perception: “I just didn’t know what to believe”

In the context of weak disbelief, healthcare professionals were reported to be more open to discussing and investigating the meaning of participants’ symptoms, but the women still perceived resistance or doubt. When a healthcare professional explored the possibilities of different diagnoses, including endometriosis, this validated participants’ self-perception. However, several participants reported that when medical investigations return a result of ‘normal’ this made them question their self-perception, wondering if their symptoms were indeed normal, and therefore questioning the credibility of their own evidence. This doubt initiated a move back to a context of strong disbelief for some and a consequent decline in investigating behaviour.

‘I then got investigated for all sorts, IBS, cysts etc by the GP…I thought I was going mad’ (Janet).

‘Your mind plays tricks. I just didn’t know what to believe. You are desperate to get an answer, but then the doctor tells you nothing is wrong. This was hard to deal with’ (Alma).

### Recognising risk: “My ‘mattress moment’”

Having recognised in the context of strong belief that her life is fluctuating, in the context of weak disbelief, a woman associates her fluctuating life directly with the abnormality of her symptoms. Most participants had cycled through contexts of refusal and disbelief for a protracted period of time, oscillating between believing symptoms to be normal or abnormal dependent on their own perception of risk and the views of those from whom they sought help. For some there comes a moment of personal transition: described by Gemma as her ‘mattress moment’.

‘We were about to carry it [a blood-soiled mattress] out of the house and my poor husband is so caring. We carried this mattress out of the house and the builders were there. The builders were our friends and I felt disgusted and embarrassed. I felt so disgusted. Have I done something wrong? Is this normal? Am I not using the right stuff? Is it because I haven’t taken the tranexamic acid? You blame your body. It was only when I saw this mattress being carried out of the house that I realised how real my symptoms were. This was my “mattress moment”. (Gemma).

Gemma’s experience highlights a cease to oscillation between refusal and disbelief. Recognition of risk for this participant was not just of her symptoms, but also of her own refusal, disbelief, and empowerment. This one moment allowed her to view her situation with clarity for the first time and provided her with the self-belief to pursue a diagnosis.

### Investigating behaviour: “I would go to the doctor armed with my own research”

In the context of weak disbelief, some participants found ways to package their symptoms to demonstrate symptom credibility to healthcare professionals and enable the healthcare professional to recognise them as worthy of investigation. This approach facilitated conversations with powerful others, such as healthcare professionals, to ensure that the risk was recognised by them and actions taken. While Alma went to the doctor ‘armed with her own research,’ Vivienne used a symptom diary in her consultation with her GP:

‘They must think, is it worth pushing this girl forward for referral at such a young age? I understand this and I’m sure they were doing it in my best interests, but it also meant I was left untreated. When doctors came back to me “empty” then I would always go to the doctor armed with my own research’ (Alma).

‘Maybe she understood actually what I was experiencing wasn’t normal as I took my symptom diary in. She was impressed with it’ (Vivienne).

Even in a context of weak disbelief, taking part in a medical consultation was clearly expressed by participants as a challenging experience. These women experienced being dismissed, feeling that they were not being taken seriously and that their concerns and expectations were not valued. The impact of the clinician’s power on the woman's investigating behaviour was profound in some cases, illustrated here by Faye, who declined to seek further medical help for several years as she moved back to a context of strong disbelief:

‘So, yeah, I went to the GP and I felt so happy to get the appointment. He trampled all over my feelings and what I was telling him. No point even going as he made me feel shit. I was already dubious about going. He didn’t even acknowledge how awful things were. I didn’t go back for years’ (Faye).

#### Context of belief: “I knew my symptoms were being taken seriously”

In the belief context, the woman is validated through her experience with the healthcare professional and through a referral to gynaecology. The woman is the most empowered compared to the other contexts and believes in herself that there is a problem and participates actively in the process of obtaining a diagnosis. As they enter a context of belief with their healthcare professionals, at best the women will feel empowered, having made sense of their symptoms and experiencing confidence in the credibility of their evidence. This context focuses on being believed by others and the impact of their empowerment on women’s self-perception. A diagnosis is most likely in the belief context, and if there is any delay, it is more likely due to structural reasons (NHS waiting times).

### Power: “[I would] challenge others if they didn’t believe me or were dismissive”

All participants expressed greater power when seeking medical help in the context of belief than previously in their journey to diagnosis. A key differentiating factor in the belief context was that healthcare professionals explored the meaning of women’s symptoms with them, recognising the symptoms to be abnormal. This relates to a central narrative of being believed and feeling powerful enough to re-disclose symptoms to the healthcare professional:

‘When my doctor started to ask more questions about my symptoms and took a real interest in me as a person, I just knew that my symptoms were being taken seriously and this made me challenge others if they didn’t believe me or were dismissive. Even she said she couldn’t understand why other doctors had told me my symptoms were normal. What a moment this was for me! Yay, I was right all along! I really felt I could go through my whole story again without worrying’ (Harissa).

Even when the severity of her symptoms was not recognised by others, in the belief context Neha was empowered to challenge others’ dismissal of her symptoms, motivated by her perception of the unfairness and injustice of how her symptoms impacted her as a woman:

‘My symptoms were just getting worse and it stopped me from doing literally everything. How could this awful pain only happen to me as a woman? If anything, knowing this was a female issue, it made me push more for a diagnosis’ (Neha).

### Self-perception: “I cried as I finally started to believe in myself”

Recognition of symptoms as worthy of further investigation within the context of a clinical consultation provided participants with a sense of validation and acknowledgement that symptoms were ‘real.’

‘My God, when the GP looked through my records and saw how badly my symptoms affected me, she recognised how bad this situation was. I cried as I finally started to believe in myself and that I shouldn’t put myself down because other people don’t understand. My symptoms suddenly felt more real in a weird way’ (Faye)

Participants described validation as stemming from acknowledgement of the challenge of their personal journey or the duration of symptoms, discussion about the impact that unexplained symptoms were having on their quality of life or their relationship with their partner or a clinician taking time to guide them through the diagnostic process to rule out different potential causes of symptoms. The impact of being believed in relation to her symptoms, was a pivotal moment for Umbola:

‘I mean, even when the gynaecologist asked me how I was, I just burst into tears. It felt like someone actually believed me because they were asking me how I was feeling’ (Umbola).

Being listened to and believed facilitated acceptance of the process of investigation ahead:

‘When she explained the way referral works, I understood better that this won’t be an easy journey, but I was prepared eventually for this’ (Harissa).

### Recognising risk: “my symptoms could lead to fertility problems and cause scarring”

Participant’s perception of the risk of harm was greatest within the belief context. They began to diagnose themselves with endometriosis through self-research and online forums and pushed the healthcare professionals they encountered to facilitate diagnosis through interventions or referral to secondary care. Finding peers who had experienced similar symptoms and achieved diagnosis was a powerful factor in recognising the risk and significance of their symptoms.

‘I just had enough after years of not being believed. No way was I going to leave the GP consulting room without a way forward. I had joined an online group and all the ladies on there told me that my symptoms could lead to fertility problems and cause scarring’ (Neha)

### Investigating behaviours: “we made a plan together and slowly worked through it”

When participants were believed and felt listened to, their trust in the health professionals caring for them increased. They were less inclined to challenge and push healthcare professionals for investigations and diagnosis and described a willingness to work with the healthcare system.

‘This particular GP was wonderful. The feeling between us was so different and I knew I didn’t need to be defensive or argumentative to get a referral to the gynaecologist’ (Elisha)

The importance of trust between the patient and healthcare professional was a key factor in participants accepting the waiting times for a referral to gynaecology and further delay to definitive diagnosis. Knowing that there was an ongoing investigative pathway in progress, waiting to see a specialist did not necessarily matter:

‘I was told the wait to see a gynaecologist was long, but I knew things were moving forward and that was ok’ (Umbola)

In the belief context, Neha was able to freely discuss her symptoms, without perceiving adverse judgement from her GP, which facilitated collaborative decision-making about her care and a reframing of the balance of power in the therapeutic relationship.

‘When the GP said we need to look into the symptoms further and told me all the ways, I was happy. She wasn’t judgy and that was important to me. We made a plan together and slowly worked through it. How lovely this feeling was’ (Neha)

## Discussion

The GT generated by this study helps to explicate the contexts underpinning women’s experiences of diagnosis of endometriosis, and how they influence time to diagnosis, capturing a fluctuating life as women experience, make sense of and investigate their symptoms. The theoretical framework proposed provides a tool for professionals and those affected by endometriosis to facilitate understanding of the complex interplay of context, relationships and self-perception involved in help-seeking behaviour and ultimate diagnosis.

Delay to diagnosis of endometriosis due to procedural reasons may occur during the protracted time the medical system takes to work up to a diagnosis, for example in the process of a differential diagnosis. However, our findings highlight that avoidable social delay appears to be equally, if not more, impactful, often occurring at the hands of important others who may reassure women that their symptoms are normal, dismiss their attempts to discuss distressing symptoms as ‘making a fuss’ and actively rebuff their efforts to make sense of what is happening to them. The experience of symptom normalisation prior to seeking medical help is widely reported in the literature (Facchin et al., 2018; Grundström et al., 2016), occurring both from the perspective of self-normalisation by women and by others (Ballard, Lowton & Wright, 2006; Pugsley & Ballard, 2007; Ghai et al., 2020). Cole, Grogan and Turley (2020) highlight that self-silencing of symptoms can occur in response to the way in which other people respond or normalise symptoms, which was also strongly evident in our study. Self-normalisation of symptoms of endometriosis as an expected part of menstruation is evident in our findings, especially if the symptoms do not unduly impact their daily lives and their quality of life remains intact. This resonates with Manderson et al. (2008), who suggest that, regardless of menstrual experiences, all women will identify menstrual-related pain as normal within the context of their own life. The result of this ‘normalisation’ of symptoms in our participants was that women did not initially recognise their symptoms as abnormal, which led to many considering themselves as weak, perceiving social stigma and questioning the credibility of their own evidence, ultimately impacting negatively on their investigating behaviour and consequent time taken to achieve diagnosis.

Normalisation and dismissal of symptoms of undiagnosed endometriosis are also evident in participants’ experiences with health professionals, particularly doctors, when they attempt to make sense of and seek help for their symptoms. This finding resonates with studies in which the power dynamic between a patient and clinician is recognised through clinicians holding power through the acts of diagnosing, prescribing, referring to specialties, and treating (Whitehead, 2007; Rees, Ajjawi & Monrouxe, 2013; Nugus et al., 2010). Through interviews with 26 women with endometriosis, Young, Fisher and Kirkman (2019) identified that women are aware of the power doctors have over their wellbeing and are strongly influenced by their doctor’s views as well as their own views about their symptoms. Bontempo (2025) uses the term ‘symptom invalidation’ in her conceptual study of women with endometriosis to describe “the process by which an individual is told they are wrong in their beliefs about their symptoms and/or are told they have ulterior motives for seeking care.” Our findings highlight that the impact of ‘symptom invalidation’ can be that women with suspected endometriosis feel dismissed, disempowered and lacking in confidence in the credibility of their symptoms. Healthcare professionals, particularly doctors, should be encouraged to consider their own pre-conceptions about the symptoms with which women present and the impact these may have on their clinical reasoning and decision-making.

Healthcare professionals are often unaware of their position of power within the clinical encounter, meaning that they do not actively seek to address or mitigate it (Nimmon & Stenfors-Hayes, 2016). This is important, as our study demonstrates that when women with endometriosis experience invalidation from healthcare providers, healthcare-related behaviour can be influenced detrimentally as they may perceive this invalidation as confirming their own lack of legitimacy in seeking help, therefore moving them back to a context of strong disbelief and further delaying diagnosis, as evidenced by Faye, “I didn’t go back for years.” It is therefore vital that clinicians reflect on how they recognise and manage a potential imbalance of power in their clinical encounters with women with suspected endometriosis and act to minimise the impact of this on the outcome of their clinical consultations.

Within the primary care context, clinicians are required to consider a wide range of differential diagnoses in the process of clinical assessment (De Silva, Dixon & Vekaria, 2024) and as such, given the non specific nature of endometriosis-related symptoms, diagnosis can be challenging (Dixon et al., 2024). A recent New Zealand based study [17], found through their survey of General Practitioners that patients were referred to secondary care if primary care treatment did not work. However, the qualitative methodology of our study provides a more nuanced understanding of how women’s relational power within the clinical encounter and self-belief impacts on their health seeking behaviour, influencing clinicians’ decision making and, therefore, time taken to diagnosis. Our findings demonstrate that a clinical encounter in which a woman is listened to, believed and her challenging personal journey acknowledged, provides a sense of validation and value that enhances the credibility of a woman’s illness and legitimises both the impact of endometriosis on the adjustments that may be needed in her life and her help-seeking behaviour. To be diagnosed with endometriosis requires healthcare professional belief and validation, as they are the ‘gatekeepers’ to diagnosis and therefore hold significant power in women’s experiences of diagnosis.

When faced with unexplained symptoms of undiagnosed endometriosis, our participants initially perceived their symptoms to be at no or ‘low risk’ of causing harm to their bodies, meaning that their propensity to seek help was low, therefore delaying medical investigation and diagnosis. Moreover, it appears from our findings that when women first present to a GP with endometriosis-related symptoms, some are unlikely to have made sense of their bodily experiences and therefore may find it challenging to articulate these experiences meaningfully to a clinician. Evidence suggests that when an individual experiences a symptom, they may not recognise the symptom(s) as a problem (Smith, 2005; de Nooijer, Lechner & de Vries, 2001). Andersen et al. [3] argue that for a bodily sensation to be defined as a symptom, the individual needs to undergo a process of interpretation in relation to their social context. This notion is supported by Manderson et al. (2008) who describe the change in meaning ascribed to menstrual pain in women with endometriosis from ‘normal’ to ‘abnormal’ as engendering a more fundamental reconstruction of self and social identity. They describe this shift in meaning as a ‘circuit breaker,’ an event or experience that facilitates a change in how a woman makes sense of her menstrual symptoms, allowing her to see them as associated with abnormal pathology rather than ‘normal’ womanhood (Manderson et al., 2008). Key ‘circuit breakers’ in this qualitative study of 41 Australian women with endometrosis were identified as intercession by significant others, social disruption, biographic disruption and self-recognition (Manderson et al., 2008). These findings resonate with the experiences of participants in our study, although it was evident that the process of symptom interpretation varied for each woman and was strongly influenced by social circumstances and relationships, particularly when symptoms initially appeared during adolescence. Circuit breakers could be facilitated by healthcare professionals caring for children and young people, and pastoral teams within the school environment, by providing clear information about the symptoms of endometriosis for all young women during their teenage years. Rhandawa et al. (2021) found that the majority of adolescents in their study had no knowledge of endometriosis, resonating with our study and highlighting health literacy as key to young women recognising their bodily experiences as abnormal, even if they do not associate them with endometriosis. Open discussion and education about endometriosis by professionals would not only legitimise their experiences but may also be a catalyst in initiating help-seeking behaviour.

Our findings illustrate that the journey to diagnosis starts with women with undiagnosed endometriosis sensing that ‘something is not right’ with their body. Engman (2019) explains that for those individuals who have experienced a physical impairment since birth, this impairment will have been embodied and embedded into their sense of self over the course of their lives. However, for those women where a critical situation (such as chronic pelvic pain) occurs during their teenage years or later, then their existing embodied self will be challenged, necessitating a renegotiation of self. Embodied self is a term used to describe the inseparable unity of body and mind, emphasising the impact that a change in one can have on the other; embodiment is the way in which the embodied self engages with and experiences the world they inhabit; both concepts stem from the work of philosopher Maurice Merleau-Ponty [28]. Piran (2017) explored the experiences of embodiment in girls aged between nine and fourteen and described three ‘domains’ that influence embodiment: physical, psychological and social. Our findings highlight the impact of all three components on female embodiment, in particular how the power and influence of social relationships can impact women’s psychological response to their physical symptoms, as women move through (and oscillate between) the four contexts on their journey towards diagnosis, which often starts in their early teenage years. The disruption to the sense of embodied self experienced through the symptoms of suspected endometriosis is seen to be further exaggerated in our study by normalisation of symptoms by significant others and healthcare professionals, which leads to women questioning their own relationship with and confidence in themselves and their bodies. This experience impacts significantly on self-perception and female identity as women perceive themselves as weak for not being able to deal with ‘normal’ female reproductive processes. It is likely that consequent reduction in their perception of risk of harm from their symptoms and commitment to coping strategies that avoid recognising that they have problem might inhibit help seeking behaviours (formal or informal) and directly contribute to a delay in diagnosis.

### Strengths and limitations

The extant literature tends to focus on individual factors associated with delay to diagnosis in endometriosis, rather than how factors work together to influence investigating behaviour and subsequent diagnosis. This study provides important new insight into the complex interplay of relational, social and psychological factors influencing delay to diagnosis of endometriosis, reflecting the value of adopting the qualitative research methods used.Transferability of the findings from this study may be limited by most participants being Caucasian, only able to converse in English and recruited from the East of England. It could be argued that the sample reflected the regional population, but it is possible that the limited diversity in the sample may mean that theoretical saturation was reached earlier than might have been the case with a more diverse sample population. These factors partly relate to the study being undertaken as part of unfunded doctoral research. Although participants were aged 18 or above, thereby excluding young women under 18, all participants were able to reflect back on how their experiences changed over time, including during their adolescence.

Conducting interviews within a private room in a hospital environment may have meant that participants did not feel as comfortable to discuss their experiences as they may have done in a more neutral location or in their own homes. It is also possible that the position of the researcher as a registrar in obstetrics and gynaecology was known to the participants and this may have influenced their responses during the interviews. However, participants appeared to be sufficiently comfortable to share in-depth thoughts and reflections about their experiences, as illustrated in the verbatim quotations included. The identify of BK as both clinician and researcher may also have influenced his interpretations during data collection and data analysis, although this was anticipated by the research team and mitigations put in place, as described in the Methods section of the paper.

### Implications and future research

At present, there is no agreed definition for what constitutes ‘delay’ to diagnosis of endometriosis in terms of timeframe. Consensus should be sought from clinicians, patients and public involvement and other relevant stakeholders to clearly define delay to diagnosis. Further qualitative research to explore the delays to diagnosis in non-Caucasian populations should be considered, a recommendation supported by the findings of a pilot cross-sectional survey by Manderville et al. [27] in which black women with endometriosis identified high levels of perceived stigma and discrimination when interacting with the healthcare system.

Crucial to the process of diagnosis is the moment of vulnerability when a woman recognises their bodily experiences as abnormal and seeks medical attention for the first time. This is a critical moment, in which the clinician can have a major influence on the progression of or delay to diagnosis. It is vital, therefore, that clinicians recognise and are aware of this influential moment and the impact that they can have on a woman’s propensity to seek medical help and consequently any delay to diagnosis. Taking time to build rapport and actively listen to women will encourage them to disclose their symptoms, even if initially embarrassing, while adopting an holistic approach will allow them to express the impact of their symptoms on the quality of their life, thereby providing the clinician with greater insight to inform differential diagnosis and further investigation. This open approach to communication could also be adopted in the workplace, with awareness of endometriosis and the considerable and unpredictable impact it can have on women promoted with employers to enable them to offer appropriate support and adjustment in the working environment. Whilst menstrual education has been introduced to schools in England, further interventions are required to educate young people and schools about normal menstruation and differentiating it from abnormal.

The findings from this study help to illustrate that a woman’s first medical consultation can negatively influence her future health-seeking behaviour if she perceives healthcare professionals to be dismissive. It is therefore vital that primary care clinicians are aware of the importance of the ‘first consultation’ during which women may present with signs and symptoms of endometriosis. Clinicians have an important role in helping women to recognise the signs and symptoms of endometriosis using appropriate language and supportive resources. It is also important for clinicians in particular to have an awareness of symptom normalisation and use a biopsychosocial approach to medical history taking with women with suspected endometriosis. Whilst there is a role for self-management in patients with endometriosis [34], more supported and engaging strategies could be developed.

## Conclusions

Using CGT we were able to explore how women’s experiences of endometriosis are influenced by the contexts through which they oscillate in their journey to diagnosis of endometriosis, influenced by a complex interplay of relational power, self-perception, recognition of risk and investigating behaviour. The journey to diagnosis is multifactorial and requires sensitivity, understanding and a commitment to listen and value women’s experiences within the clinical consultation to ensure timely and appropriate investigation and management.

## Supplementary Information

Supplementary Material 1.

## Data Availability

The datasets used and/or analysed during the current study are available from the corresponding author on reasonable request.

## References

[1] Agarwal SK, Chapron C, Giudice LC, Laufer MR, Leyland N, Missmer SA, et al. Clinical diagnosis of endometriosis: a call to action. Am J Obstet Gynecol. 2019Apr;220(4):354.e1-354.e12. 10.1016/j.ajog.2018.12.039 30625295

[2] All Party Parliamentary Group (APPG) on Endometriosis. Endometriosis in the UK: time for change. 2020. Available at: https://www.endometriosis-uk.org/sites/default/files/files/Endometriosis%20APPG%20Report%20Oct%202020.pdf [Accessed 20 December 2024]

[3] Andersen RS, Vedsted P, Olesen F, Bro F, Søndergaard J. Patient delay in cancer studies: a discussion of methods and measures. BMC Health Services Research. 2009 Oct 19;9(1). 10.1186/1472-6963-9-189 PMC2770466 19840368

[4] Ballard K, Lowton K, Wright J. What’s the delay? A qualitative study of women’s experiences of reaching a diagnosis of endometriosis. Fertil Steril. 2006Nov;86(5):1296–301. 17070183 10.1016/j.fertnstert.2006.04.054

[5] Birks M, Mills J. Grounded Theory: A Practical Guide. SAGE; 2011.

[6] Bontempo AC. Conceptualizing Symptom Invalidation as Experienced by Patients With Endometriosis. Qual Health Res. 2025Aug 8;35(2):248–63. 39116403 10.1177/10497323241253418

[7] Bullo S. “I feel like I’m being stabbed by a thousand tiny men”: The challenges of communicating endometriosis pain. Health: An Interdisciplinary Journal for the Social Study of Health, Illness and Medicine. 2019 Feb 19;24(5):476–92. 10.1177/1363459318817943 30782020

[8] Carter MJ, Fuller C. Symbolic interactionism. Sociopedia. 2015. 10.1177/205684601561.

[9] Charmaz K. Constructing Grounded Theory: A Practical Guide Through Qualitative Analysis. SAGE; 2006.

[10] Cole JM, Grogan S, Turley E. “The most lonely condition I can imagine”: Psychosocial impacts of endometriosis on women’s identity. Fem Psychol. 2020Jun 23;31(2):171–91.

[11] Conlon C, Timonen V, Elliott-O’Dare C, O’Keeffe S, Foley G. Confused About Theoretical Sampling? Engaging Theoretical Sampling in Diverse Grounded Theory Studies. Qualitative Health Research. 2020 Jan 20;30(6):947–59. 10.1177/1049732319899139 31959073

[12] Davenport S, Smith D, Green D. Barrriers to a timely diagnosis of endometriosis: a qualitative systematic review. Obstetrics & Gynecology. 2023 Sept; 142(3):571–583. 10.1097/AOG.0000000000005255 37441792

[13] de Nooijer J, Lechner L, de Vries H. A qualitative study on detecting cancer symptoms and seeking medical help; an application of Andersen’s model of total patient delay. Patient Educ Couns. 2001Feb;42(2):145–57. 11118780 10.1016/s0738-3991(00)00104-x

[14] De Silva PM, Dixon S, Vekaria G. Restructuring endometriosis care. BMJ. 2024Nov;18: q2416. 10.1136/bmj.q2416 39557460

[15] Dixon S, McNiven A, Talbot A, Hinton L. Navigating possible endometriosis in primary care: a qualitative study of GP perspectives. Br J Gen Pract. 2021May 4;71(710):e668–76. 33950856 10.3399/BJGP.2021.0030 PMC8340732

[16] Dragan IM, Isaic-Maniu A. An Original Solution for Completing Research through Snowball Sampling—Handicapping Method. Advances in Applied Sociology. 2022;12(11):729–46.

[17] Ellis K, Meador A, Ponnampalam A, Wood R. Survey of General Practitioner Perspectives on Endometriosis Diagnosis, Referrals, Management and Guidelines in New Zealand. Health Expectations. 2024 Sep 2;27(5). 10.1111/hex.70015 PMC11369013 39223834

[18] Engman A. Embodiment and the foundation of biographical disruption. Soc Sci Med. 2019Mar;225:120–7. 30825759 10.1016/j.socscimed.2019.02.019

[19] Gaedtke A. Diagnosis, Literature, and Legitimation. Am Lit Hist. 2023Jun 21;35(3):1317–25.

[20] Gallagher JS, DiVasta AD, Vitonis AF, Sarda V, Laufer MR, Missmer SA. The Impact of Endometriosis on Quality of Life in Adolescents. J Adolesc Health. 2018Dec;63(6):766–72. 30454733 10.1016/j.jadohealth.2018.06.027

[21] Ghai V, Jan H, Shakir F, Haines P, Kent A. Diagnostic delay for superficial and deep endometriosis in the United Kingdom. J Obstet Gynaecol. 2020Jul 22;40(1):83–9. 31328629 10.1080/01443615.2019.1603217

[22] Giudice LC, Kao LC. Endometriosis. The Lancet. 2004Nov;364(9447):1789–99. 10.1016/S0140-6736(04)17403-5 15541453

[23] Grundström H, Kjølhede P, Berterö C, Alehagen S. “A challenge” – healthcare professionals’ experiences when meeting women with symptoms that might indicate endometriosis. Sexual & Reproductive Healthcare. 2016Mar;7:65–9. 26826048 10.1016/j.srhc.2015.11.003

[24] Hallberg L. The, “core category” of grounded theory: making constant comparisons. Int J Qual Stud Health Well Being. 2006;1(3):141–8.

[25] Karavadra B, Simpson P, Prosser-Snelling E, Mullins E, Stöckl A, Morris E. A Study to Explore the Impact of Endometriosis in the United Kingdom: A Qualitative Content Analysis. Gynecology and Obstetrics Research– Open Journal. 2019 Dec 30;6(1):11–9.

[26] Lightbourne A, Foley S, Dempsey M, Cronin M. Living With Endometriosis: A Reflexive Thematic Analysis Examining Women’s Experiences With the Irish Healthcare Services. Qualitative Health Research. 2023 Nov 21;34(4):311–22. Manderson L, Warren N, Markovic M. Circuit breaking: pathways of treatment seeking for women with endometriosis in Australia. Qualitative Health Research. 2008;18(4):522–534

[27] Manderville J, Pollack AZ, Kornegay L, Gupta J. Stigma and discrimination experienced by Black women with endometriosis in the Washington, DC, Metropolitan area: A pilot of the ENDO-served study. Int J Gynecol Obstet. 2025;00:1–3. 10.1002/ijgo.70042. 10.1002/ijgo.70042 40022574

[28] Merleau-Ponty M. Phenomenology of Perception. Routledge; 1945.

[29] Moss KM, Doust J, Homer H, Rowlands IJ, Hockey R, Mishra GD. Delayed diagnosis of endometriosis disadvantages women in ART: a retrospective population linked data study. Hum Reprod. 2021Oct 5;36(12):3074–82. 34610108 10.1093/humrep/deab216

[30] National Institute for Health and Care Excellence (NICE) (2017; updated 2024) Endometriosis: diagnosis and management: NG73. https://www.nice.org.uk/guidance/ng73 [Accessed 12 Dec 2024]

[31] Nimmon L, Stenfors-Hayes T. The “Handling” of power in the physician-patient encounter: perceptions from experienced physicians. BMC Medical Education. 2016 Apr 18;16(1). 10.1186/s12909-016-0634-0 PMC4835893 27091146

[32] Nisolle M, Donnez J. Reprint of: Peritoneal endometriosis, ovarian endometriosis, and adenomyotic nodules of the rectovaginal septum are three different entities. Fertil Steril. 2019Oct;112(4):e125–36. 31623724 10.1016/j.fertnstert.2019.08.081

[33] Nugus P, Greenfield D, Travaglia J, Westbrook J, Braithwaite J. How and where clinicians exercise power: Interprofessional relations in health care. Soc Sci Med. 2010Sep;71(5):898–909. 20609507 10.1016/j.socscimed.2010.05.029

[34] O’Hara R, Roufeil L. Self-Management Among People Living With Endometriosis: A Qualitative Study. Qualitative Health Research. 2024 Nov 14; 10.1177/10497323241291787 PMC12308033 39540787

[35] Piran N. The Developmental Theory of Embodiment. In: Journeys of Embodiment at the Intersection of Body and Culture. London: Elsevier; 2017

[36] Pugsley Z, Ballard K. Management of endometriosis in general practice: the pathway to diagnosis. Br J Gen Pract. 2007Jun;57(539):470–6. 17550672 PMC2078174

[37] Randhawa AE, Tufte-Hewett AD, Weckesser AM, Jones GL, Hewett FG. Secondary School Girls’ Experiences of Menstruation and Awareness of Endometriosis: A Cross-Sectional Study. J Pediatr Adolesc Gynecol. 2021Oct;34(5):643–8. 33548448 10.1016/j.jpag.2021.01.021

[38] Rees CE, Ajjawi R, Monrouxe LV. The construction of power in family medicine bedside teaching: a video observation study. Med Educ. 2013Jan 16;47(2):154–65. 23323654 10.1111/medu.12055

[39] Roomaney R, Kagee A. Coping strategies employed by women with endometriosis in a public health-care setting. J Health Psychol. 2016Jul 10;21(10):2259–68. 25769875 10.1177/1359105315573447 PMC4568158

[40] Smith EM. Telephone interviewing in healthcare research: a summary of the evidence. Nurse Res. 2005Jan;12(3):32–41. 15793975 10.7748/nr2005.01.12.3.32.c5946

[41] Taylor HS, Kotlyar AM, Flores VA. Endometriosis is a chronic systemic disease: clinical challenges and novel innovations. The Lancet. 2021Feb 27;397(10276):839–52. 10.1016/S0140-6736(21)00389-5 33640070

[42] Tie CY, Birks M, Francis K. Grounded theory research: A design framework for novice researchers. SAGE Open Medicine. 2019 Jan;7. 10.1177/2050312118822927 PMC6318722 30637106

[43] Lancet T. Endometriosis: addressing the roots of slow progress. The Lancet. 2024Oct;404(10460):1279. 10.1016/S0140-6736(24)02179-2 39368831

[44] Whitehead C. The doctor dilemma in interprofessional education and care: how and why will physicians collaborate? Med Educ. 2007Oct;41(10):1010–6. 17908118 10.1111/j.1365-2923.2007.02893.x

[45] Young K, Fisher J, Kirkman M. Partners instead of patients: Women negotiating power and knowledge within medical encounters for endometriosis. Fem Psychol. 2019Jan 30;30(1):22–41.

