# Supplementary figures and images for: ‘I don’t know what normal has been’: a grounded theory exploration of the journey to endometriosis diagnosis
Source: BMC Womens Health. 2025 Jul 4;25:319. doi: 10.1186/s12905-025-03869-y (PMC12232144; doi:10.1186/s12905-025-03869-y)

**Supplementary File 1: Interview topic guide- participants with endometriosis**


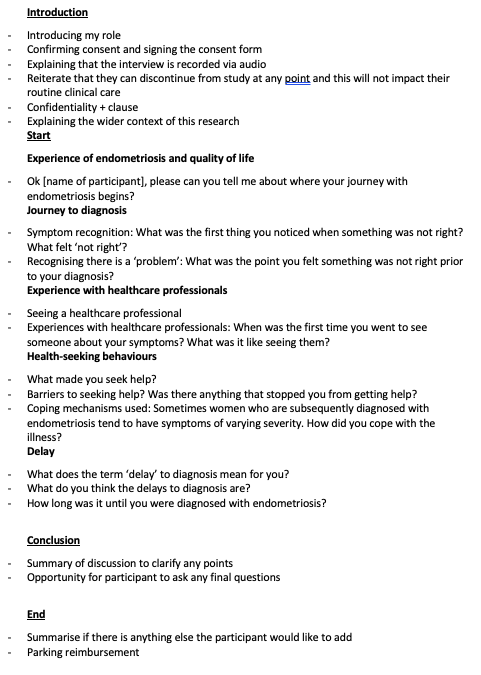

Supplement: Supplementary file 1 — Supplementary Material 1. [file 12905_2025_3869_MOESM1_ESM.docx]
